# Insight into Single-Molecule Imaging Techniques for the Study of Prokaryotic Genome Maintenance

**DOI:** 10.1021/cbmi.4c00037

**Published:** 2024-06-18

**Authors:** Nischal Sharma, Antoine M. van Oijen, Lisanne M. Spenkelink, Stefan H. Mueller

**Affiliations:** 1 Molecular Horizons and School of Chemistry and Molecular Bioscience, University of Wollongong, Wollongong, New South Wales 2522, Australia

**Keywords:** Super-resolution microscopy, In vitro single-molecule microscopy, In vivo single-molecule microscopy, Single-molecule approach, Genome maintenance, DNA replication, Transcription, DNA repair, Recombination, Fluorescence microscopy, Atomic force microscopy

## Abstract

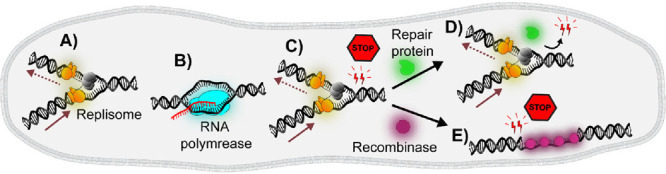

Genome maintenance comprises a group of complex and interrelated processes crucial for preserving and safeguarding genetic information within all organisms. Key aspects of genome maintenance involve DNA replication, transcription, recombination, and repair. Improper regulation of these processes could cause genetic changes, potentially leading to antibiotic resistance in bacterial populations. Due to the complexity of these processes, ensemble averaging studies may not provide the level of detail required to capture the full spectrum of molecular behaviors and dynamics of each individual biomolecule. Therefore, researchers have increasingly turned to single-molecule approaches, as these techniques allow for the direct observation and manipulation of individual biomolecules, and offer a level of detail that is unattainable with traditional ensemble methods. In this review, we provide an overview of recent in vitro and in vivo single-molecule imaging approaches employed to study the complex processes involved in prokaryotic genome maintenance. We will first highlight the principles of imaging techniques such as total internal reflection fluorescence microscopy and atomic force microscopy, primarily used for in vitro studies, and highly inclined and laminated optical sheet and super-resolution microscopy, mainly employed in in vivo studies. We then demonstrate how applying these single-molecule techniques has enabled the direct visualization of biological processes such as replication, transcription, DNA repair, and recombination in real time. Finally, we will showcase the results obtained from super-resolution microscopy approaches, which have provided unprecedented insights into the spatial organization of different biomolecules within bacterial organisms.

## 1 Introduction

**Figure 1:**
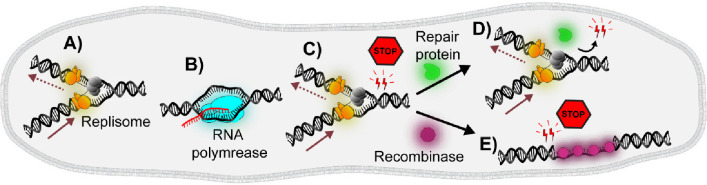
Schematics of genome maintenance processes. (A) DNA replication: The helicase (gray) unwinds DNA, while the DNA polymerases (orange) synthesize DNA continuously on the leading strand (continuous arrow) and discontinuously on the lagging strand in the opposite direction (discontinuous arrow). (B) Transcription: RNA polymerase (cyan) transcribes RNA (indicated in red), forming a transcription elongation complex (C) DNA damage, caused by UV radiation, chemical agents, or nucleoid-associated proteins, induces alterations in the structure of DNA, impeding processes such as DNA replication. (D) DNA repair proteins (green) are recruited to the site of damaged DNA, or (E) DNA recombination proteins (magenta) bound to single-strand DNA intermediates, are involved in DNA damage repair to maintain genetic integrity within the cell.

Genomic maintenance comprises the complex cellular processes that cells employ to safeguard the integrity and stability of their genetic material. In prokaryotes, these processes play a crucial role in the development of antibiotic resistance.^1−3^ Antibiotic resistance is one of the biggest health threats of this century.^4,5^ To combat antibiotic resistance, a thorough understanding of prokaryotic genomic maintenance is crucial. Prokaryotic genome maintenance is achieved through a series of complex processes, crucial for the duplication and survival of cells.^6^ These processes preserve the integrity of the genetic material, which, in prokaryotic cells, is housed within the small, circular DNA molecule found in the nucleoid region.^7−10^ Genome maintenance involves events throughout the cell cycle such as DNA replication,^11,12^ where the genetic material is duplicated with precision, and transcription and translation,^13,14^ where the information encoded in the DNA is transcribed into RNA and translated into functional proteins (Figure 1A and 1B).^7,15−17^ However, the genome of all living organisms is constantly subject to damage. This damage occurs either through external factors such as exposure to toxic chemicals or radiation, or due to disruptions in fundamental cellular processes such as DNA replication and transcription (Figure 1C). Cells have DNA repair and recombination mechanisms to respond to these damages and to ensure the fidelity of the genomic information within cells.^18^ These mechanisms detect and repair DNA lesions, preventing the accumulation of mutations that could compromise genome integrity (Figure 1D and 1E). Understanding the behavior and interactions of individual biomolecules involved in genome maintenance pathways, provides a crucial piece of information about the complex molecular mechanisms that govern these cellular processes.

While traditional ensemble methods have been invaluable in the study of genome maintenance for decades, they fall short in providing a detailed description of the real-time activities. This is because ensemble-average analysis will not pinpoint the subpopulation of biomolecules undergoing transient interactions within the heterogeneous population.^19^ Such transient interactions, which are often overlooked by ensemble analysis, could be crucial for understanding the mechanisms underlying genome maintenance. They could provide valuable information about additional mechanistic details of antibiotic resistance mechanisms, which could be vital for the development of therapeutic drugs. In contrast, single-molecule approaches provide the ability to study heterogeneity with high spatial and temporal resolution, and allow the observation of rare and transient events. The development of this approach can be traced back to the early 1960s, when researchers first began to use a microscope to study single molecules.^20^ Since then, the field has expanded to include a wide range of single-molecule techniques with applications in both in vitro and in vivo systems, to further our understanding of complex biological processes of genome maintenance.

Single-molecule methods can roughly be divided into two classes: force-based techniques and imaging techniques. The use of force-based techniques has been reviewed elsewhere.^21,22^ In this review, we will focus on recent single-molecule imaging techniques employed in in vitro and in vivo studies of complex genome maintenance processes within prokaryotic organisms. First, we will explore the principles of in vitro single-molecule imaging techniques, including TIRF microscopy and AFM. Next, we will discuss in vivo imaging approaches, such as highly inclined and laminated optical sheet (HILO, also known as near TIRF) microscopy, as well as advanced super-resolution microscopy techniques, including Stimulated Emission Depletion Microscopy (STED), Single-Molecule Localization Microscopy (SMLM), Point Accumulation for Imaging in Nanoscale Topography (PAINT), and Structured Illumination Microscopy (SIM). We will then showcase studies that have applied these single-molecule approaches to visualize genome maintenance processes such as DNA replication, transcription, DNA repair, and recombination. Finally, we will discuss the insights gained from super-resolution microscopy techniques, which have enabled researchers to explore the nanoscale architecture of cellular components and their interactions, providing valuable insights into the complex mechanisms governing genome stability.

## 2 Overview of Imaging Techniques

### 2.1 In Vitro Single-Molecule Fluorescence Imaging Techniques

In vitro single-molecule studies allow examination of individual molecules or molecular assemblies, such as purified proteins, nucleic acids, or other biomolecules, outside of their natural biological context, typically within a controlled laboratory environment. These studies enable precise manipulation and observation of molecule behavior, free from the complexities of cellular systems. Here, we focus on the principles of fluorescence-based TIRF and AFM in vitro single-molecule approaches.

#### 2.1.1 Total Internal Reflection Fluorescence (TIRF) Microscopy

**Figure 2:**
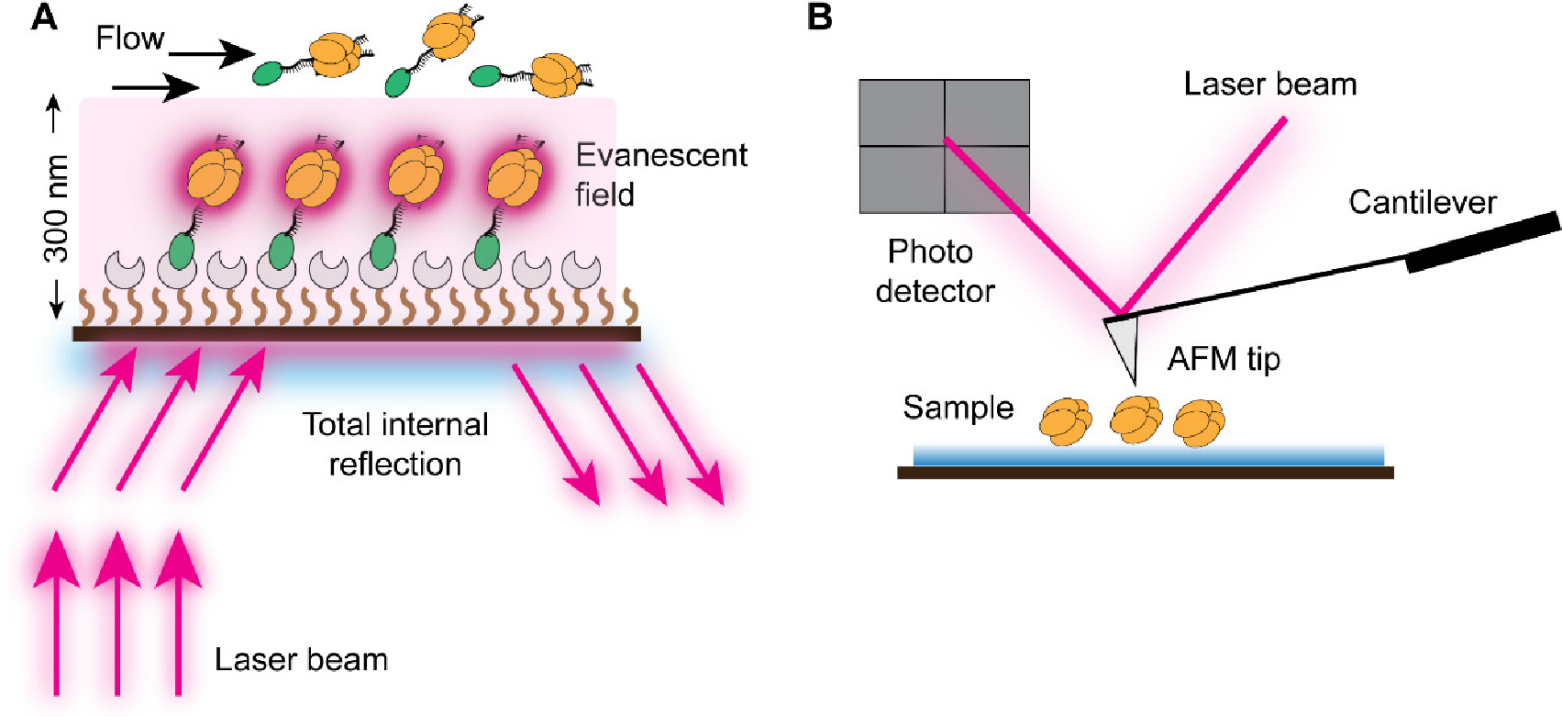
In vitro single-molecule fluorescent imaging techniques. (A) TIRF: Fluorescently labeled molecules excited by the evanescent wave enable the real-time visualization of molecules localized near the interface with high sensitivity and minimal background noise from the bulk solution. (B) AFM: A laser beam directed onto the back of the cantilever with a sharp tip is deflected as the probe tip scans the surface. The changes in the deflections are detected by the photodetector and can be used to generate a 3-D image of the sample surface.

TIRF microscopy is one of the most frequently used imaging techniques since the late 1990s enabling high-contrast visualization of fluorophores near the sample surface. The principle of TIRF microscopy is based on the phenomenon of total internal reflection and the evanescent wave generated at the interface between two media with different refractive indices (Figure 2A).^23^ Total internal reflection (TIR) occurs when light traveling through a medium with a higher refractive index (e.g., glass or quartz) hits the interface with a lower refractive index (e.g., water or air) at an angle greater than the critical angle.^23^ When this happens, light is internally reflected within the higher refractive index medium instead of being transmitted through the interface. At the interface where TIR occurs, an evanescent wave with a limited penetration depth (typically around 100–300 nanometers) is formed into the lower refractive index medium.^23−25^ The evanescent wave, confined to the surface, selectively excites fluorophores near the interface, resulting in improved axial resolution. Only molecules close to the surface contribute to the fluorescence signal, reducing background noise. This enhanced signal-to-noise ratio produces high-contrast images of selectively illuminated fluorophore samples.^23,24^ In the context of prokaryotic genome maintenance, TIRF microscopy has been widely employed to visualize DNA replication,^26−30^ transcription,^31,32^ translation,^32^ DNA repair, and recombination,^33^ and cell segregation during the bacterial cell cycle in real-time.^34^

To study individual molecules using TIRF microscopy, the molecules of interest must be fluorescently labeled and immobilized on a surface. A wide range of fluorescent molecules have been developed, spanning from fluorescent proteins that can be genetically fused to proteins of interest^35,36^ to synthetic dyes^37^ and nanoparticles (Quantum-dots),^38,39^ which can be tuned to exhibit the desired spectral properties.^40^ Once the molecules are fluorescently labeled and immobilized, they can be excited selectively near the surface through TIRF microscopy, enabling real-time imaging and analysis of their behavior and interactions.

Since in TIRF microscopy only molecules close to the surface are illuminated, immobilization of the molecules of interest is necessary to image biological reactions for prolonged periods of time. Various strategies have been devised to achieve specific tethering of molecules of interest while minimizing nonspecific adhesion as well as fluorescent background. A common strategy is to coat the surface with high-molecular-weight polyethylene glycol (PEG), a small fraction of which contains biotin-moieties. PEG can be adsorbed to glass surfaces, either covalently via amine-reaction cross-linking following amino-silane treatment of glass, or nonspecifically using a copolymer consisting of poly-l-lysine-biotin-PEG.^41^

Alternatively, surfaces can be treated with dichlorodimethylsilane. Biotinylated bovine serum albumin is then nonspecifically adsorbed to this surface. Further nonspecific protein adsorption is inhibited by treatment with the nonionic detergent Tween-20.^42^ The biotin-containing surface can then be coated with streptavidin, which in turn allows for specific immobilization of any biotin-containing biomolecule. Biotinylated DNA or RNA oligonucleotides are commercially available and can readily be ligated to or incorporated into longer sequences, using for example PCR-reactions including biotinylated nucleotides or primers.^43,44^ Proteins can be biotinylated either by genetically encoding a biotin-acceptor domain and treating or coexpressing with a biotin protein ligase^45,46^ or using biotin derivatives that react with primary amines.^47^ More detailed protocols for immobilization as well as fluorescent labeling of proteins and nucleic acids is reviewed in more detail elsewhere.^48^

In addition, TIRF microscopy combined with single-molecule Förster Resonance Energy Transfer (smFRET) is another powerful technique used to study conformational changes and dynamics of individual molecules at the single-molecule level. During FRET, energy is transferred nonradiatively between two fluorophores (donor and acceptor) when they are in close proximity, typically within a range of 1 to 10 nanometers.^49^ In smFRET experiments, biomolecules are tagged with donor and acceptor fluorophores with spectral overlap, such as Cy3 and Cy5. When the donor fluorophore is excited, energy can be transferred to the nearby acceptor fluorophore, resulting in light emission at the acceptor fluorophore’s emission wavelength.^49,50^ The efficiency of this energy transfer is highly sensitive to the distance between donor and acceptor fluorophores and can therefore be used to measure distances between 1–10 nm. smFRET is used to study protein or nucleic acid interactions, conformational changes, stoichiometry, and intramolecular distances of biomolecules in real-time, which have been well reviewed elsewhere.^51−54^

#### 2.1.2 Atomic Force Microscopy (AFM)

AFM is a nanoscale imaging and manipulation technique that employs a sharp tip mounted on a cantilever to scan the surface of a sample. As the tip interacts with atoms on the sample surface at *x* and *y* positions, the resulting forces cause the cantilever, measuring the height (*z*), to bend (Figure 2B).^21^ The deflection of the cantilever, which oscillates close to its mechanical resonance frequency, is then measured using a laser beam reflected off the back of the cantilever onto a position-sensitive photodetector. This enables the creation of a detailed topographic map of the sample’s surface under physiological conditions.^55^ AFM operates in dynamic imaging modes, such as tapping mode or noncontact mode, which minimize the interaction forces between the tip and the sample surface. It provides crucial structural and functional information on protein–DNA interactions at the molecular level, governing biomolecular processes such as DNA replication,^56,57^ transcription,^58−60^ repair,^61−64^ and recombination.^65^ The tapping mode of AFM allows the preservation of the structural integrity of delicate biological samples, facilitating their prolonged visualization for up to several hours.^66^ However, conventional AFM’s limitation of capturing only one image every few minutes proves inadequate time resolution for the visualization of rapid biological processes, which often occur at subsecond time scales. Recently, these studies have been extended with the use of high-speed AFM (HS-AFM), enabling the visualization of dynamic behaviors in DNA, proteins, and protein–DNA complexes at the millisecond time scale resolution.^67^

The introduction of HS-AFM in 2001 marked a significant breakthrough, enabling the observation of dynamic behaviors in biological molecules.^68^ HS-AFM operates at significantly higher scanning speeds compared to conventional AFM techniques. While traditional AFM might require seconds or even minutes to acquire an image, HS-AFM can capture images in real-time or at rates of 10–16 frames per second.^69^ HS-AFM operates on the same basic principles as traditional AFM but with modifications to enable faster imaging. The operational principle of HS-AFM involves the miniaturization of the moving components of traditional AFM, mainly the cantilever and scanner, allowing for faster response times and rapid scanning across the sample surface.^68,70^ It is accomplished by optimizing the design and reducing the mass of the moving parts, allowing for swift responses to changes in the sample surface. Despite the gain in speed, the new probe is engineered in a way that does not compromise its overall performance, ensuring that the probe remains effective for capturing high-resolution images. Due to these powerful features, HS-AFM offers a unique ability to concurrently evaluate the structure and dynamics of individual protein molecules in motion, such as the walking of myosin V along actin filaments.^71^ Studies using HS-AFM have been well reviewed by Ando et al. 2013, Uchihashi et al. 2016, Ando, 2017, Fukuda and Ando, 2023.^69,70,72,73^

### 2.2 In Vivo Single-Molecule Fluorescence Imaging Techniques

In vivo single-molecule studies involve the examination of individual molecules within living cells, allowing researchers to examine the biological processes within their native environment. These studies often employ single-molecule fluorescence microscopy techniques such as HILO or advanced imaging techniques such as super-resolution microscopy to visualize molecules within cells or tissues. Unlike in vitro imaging techniques, in vivo imaging does not allow for precise control of the reaction conditions. However, in vivo imaging provides a clearer understanding of how individual biomolecules behave in their natural environment.

#### 2.2.1 Highly Inclined and Laminated Optical Sheet (HILO)

**Figure 3:**
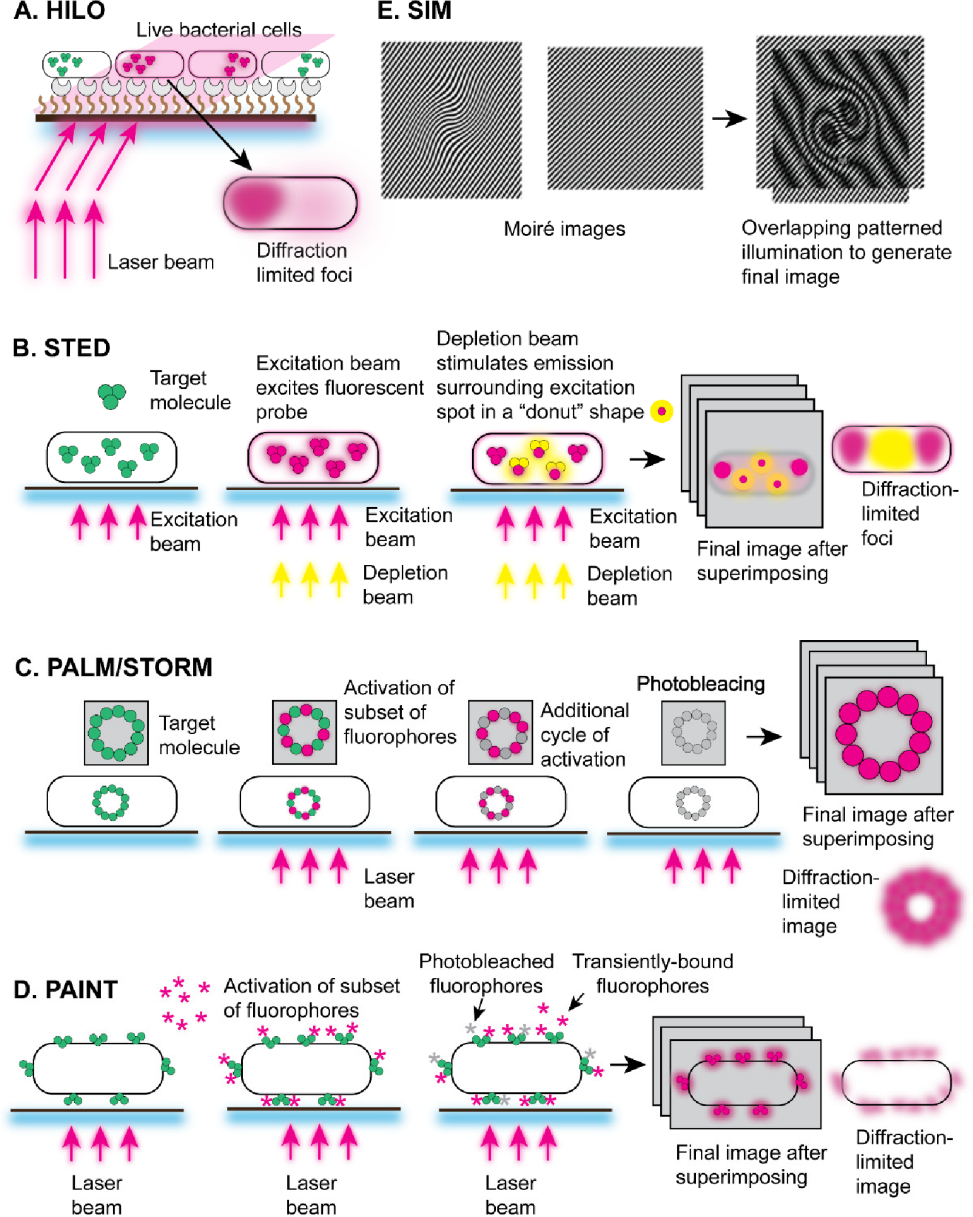
In vivo single-molecule fluorescent imaging techniques. (A) HILO: The laser excitation is directed onto the sample (for example, bacterial cells) at a highly inclined angle relative to the optical axis of the microscope, causing most of the fluorescence emission to be emitted above the focal plane of the objective lens. The out-of-focus fluorescence is greatly reduced, resulting in improved image contrast and optical sectioning (B-E) Super-resolution techniques. (B) STED: Two laser beams are used in this approach, an excitation beam that excites fluorophores and a depletion beam (or STED beam) that de-excites fluorophores forming a “donut” shape surrounding the excitation spot. This effectively reduces the size of the PSF and enables resolution beyond the diffraction limit. (C) PALM/STORM: By activating and imaging sparse subsets of molecules tagged with photoactivatable or photoswitchable dyes, their exact positions can be determined with high precision in each frame. Steps of activation, imaging, and localization are repeated iteratively until a sufficient number of molecules have been imaged to reconstruct the super-resolved image. (D) PAINT: The fluorescent PAINT probes, applied to the sample such as fixed cells, transiently bind to their complementary targets or undergo photobleaching. Super-resolved images can be constructed by repeatedly imaging the same region of interest and accumulating the positions of fluorescent spots over time, achieving resolution beyond the diffraction limit of light. (E) SIM: SIM projects a pattern of structured light onto the sample and generates Moiré patterns by interacting with the sample’s features. By capturing multiple images with varied pattern orientations, SIM extracts high-resolution details beyond the diffraction limit using computational algorithms. Reproduced with permission from ref (82). Copyright 2005 National Academy of Sciences.

The principle of the HILO technique involves the creation of a thin, highly inclined sheet of light that illuminates a specimen (Figure 3A).^74^ Similarly to TIRF microscopy, this illumination is achieved by directing a laser beam through a high numerical-aperture (NA) objective lens. In contrast to TIRF, the laser beam is adjusted to hit the coverslip–water interface at an angle slightly below the critical angle for total internal reflection. By additionally minimizing the illuminated area a thin optical section is created within the specimen, selectively exciting fluorescence only within this illuminated plane while minimizing out-of-focus background fluorescence. The thin optical section obtained with HILO allows for high-resolution, three-dimensional imaging of biological samples with minimal disturbance to the specimen. Unlike TIRF microscopy, which can only probe the bottommost ∼100 nm of the cell surface and is unsuitable for imaging the inside of cells, HILO offers enhanced contrast and greater imaging depth capacity, making it more suitable for single-molecule studies within living systems. Given its unique features, HILO enables precise localization and tracking of fluorescently labeled proteins within subcellular compartments,^75−77^ thereby facilitating the study of protein trafficking, localization, and turnover dynamics. This technique has found widespread application in the study of subpathways of DNA repair and recombination processes within bacterial cells.^33^

#### 2.2.2 Super-Resolution Microscopy

Super-resolution microscopy has emerged as a revolutionary tool in the realm of biological imaging. This advanced imaging technique has surpassed the conventional resolution limits of light microscopy well beyond 200 nanometers, providing unprecedented insights into the nanoscale architecture of biomolecules.^78−81^ In 2014, the Nobel Prize in Chemistry was awarded to Eric Betzig, Stefan Hell, and William E. Moerner for their pioneering work in developing super-resolved fluorescence microscopy. The applications of super-resolution microscopy span a wide range of biological studies, from unraveling the complexities of cellular organelles to tracking individual biomolecules in living cells. Notably, these techniques are predominantly employed in live-cell imaging due to their rapidity and 3D imaging capabilities. However, it must be noted that these techniques mostly provide spatiotemporal information about different cellular components rather than offering real-time kinetic information about biological processes associated with genome maintenance within the cell. Here, we focus on the principles of four super-resolution microscopy techniques: STED, SMLM, PAINT, and SIM (Figure 3B–E). The results obtained from the application of these techniques will be discussed in section 3.

##### 2.2.2.1 STED (Stimulated Emission Depletion)

The principle of STED involves the controlled deactivation of fluorophores in a sample using two synchronized laser beams: the excitation beam and the de-excitation or STED beam.^80,83^ First, a fluorescent probe within the sample is excited by the excitation beam from the ground state, and then it is de-excited or depleted by the second STED beam, via stimulated emission (SE), within a few nanoseconds after the excitation event. When the STED beam interacts with the excited fluorophores, it induces stimulated emission in a donut shape that surrounds the excitation spot. This stimulated emission effectively depletes the fluorophores from the outer region, leaving only the central region in an excited state. By depleting the excited fluorophores in the outer region through stimulated emission, the effective size of the excitation spot is reduced. This reduction in the size of the excitation spot allows for imaging with a spatial resolution beyond the diffraction limit (Figure 3B). These methods are primarily used for visualization and precise localization of intracellular structures such as cell division proteins,^84,85^ allowing researchers to study their morphology, organization, and dynamics with unprecedented resolution.

##### 2.2.2.2 SMLM (Single-Molecule Localization Microscopy)

PALM and STORM are both are SMLM techniques used for super-resolution imaging beyond the diffraction limit of light. While they share similarities in their principles, there are some key differences between them. The principle of PALM involves the use of photoactivatable fluorescent proteins fused to target proteins of interest within cells.^79^ These photoactivatable fluorescent proteins are initially in a nonfluorescent state. By using a brief pulse at a specific wavelength of light, a sparse subset of fluorescent proteins can be activated, while the rest remain dark. Ideally only a single fluorophore is visible within a diffraction limited volume at any time. The position of this molecule can then be determined with a precision below the diffraction limit.^86,87^ Through repeated cycles of stochastic activation and imaging, a super-resolved image is gradually constructed. This provides nanoscale details of the distributions of proteins within the cellular ultrastructure over time (Figure 3C).^79^ PALM requires low laser powers to activate and excite fluorophores and does not require an imaging buffer, which could be toxic, making it the most suitable method for live-cell imaging. However, the efficiency of labeling and expression of the fluorescent proteins within cells can impair accurate quantification or imaging when using PALM techniques.

STORM is based on the principle of single-molecule localization of subsets of photoswitchable organic fluorophores and photoswitching buffer.^78,88^ These fluorophores can be switched between an active and inactive state. Like in PALM, only a small subset of fluorophores is activated at a time, and their precise positions are determined through localization. After localization, the activated fluorophores are deactivated, allowing another subset to be activated. The final image is reconstructed from the accumulated localization data, providing a super-resolved image of the sample well beyond the diffraction limit, down to tens of nanometers.^78^ However, STORM imaging is typically carried out on fixed-cell samples because implementing it in live-cell imaging is challenging due to the requirement for precisely controlled imaging conditions. Cellular motions, photobleaching, and background noise can interfere with the stochastic switching of fluorophores, making it difficult to achieve reliable super-resolution imaging in real-time. Recently, direct STORM (dSTORM) has become a widely used form of SMLMs,^88,89^ implemented for imaging live-cells.^90,91^ dSTORM utilizes conventional organic dyes like Cy3, Cy5, Alexa Fluor dyes, and ATTO dyes, which are either linked to antibodies or attached separately to the target molecule. Researchers have employed SMLM to study dynamic biological processes, including tracking DNA-binding proteins,^92−94^ chromosomal segregation,^95,96^ and chromosome organization by nucleoid-associated proteins^97^ within live bacterial cells with unparalleled precision.

##### 2.2.2.3 PAINT (Point Accumulation for Imaging in Nanoscale Topography)

Unlike PALM and STORM methods, which face limitations due to the photobleaching of dye molecules, PAINT-based methods benefit from a continuous supply of fluorescent probes. This continuous replenishment facilitates longer imaging times. A fluorescent label transiently binds to the target biomolecule and becomes momentarily immobilized, leading to increased localization density and the potential for achieving high-resolution images up to 25 nm (Figure 3D).^98^ This approach has been particularly powerful in the study of the dynamics of cell membranes with high spatial and temporal resolution.^99,100^

##### 2.2.2.4 SIM (Structured Illumination Microscopy)

Unlike STED, SIM does not rely on laser scanning microscopes, but rather enhances the resolution of common wide-field microscopes by using patterned illumination. The resulting images are superpositions of the known illumination pattern and the unresolved structure of the biological sample. If the spatial frequency of the introduced pattern approaches the spatial frequencies present in the sample, then large-scale interference patterns (so-called Moiré fringes) are created (Figure 3E).^101^ The Moiré fringes are much coarser than the initial sample and can easily be resolved. Yet they contain all the information about the initial sample. Sophisticated computational algorithms are then applied to deconvolve the images leading to ∼ 2-fold improvements in resolution. Sample preparation applicable for standard fluorescence microscopy can be readily employed for SIM without requiring additional effort.^102^ Additionally, SIM can be applicable in multicolor imaging, typically accommodating up to four distinct color channels. It does not require any specific fluorophores for sample labeling, except for a reasonable resistance to photobleaching.^102^ Furthermore, SIM can be readily combined with other widefield super-resolution techniques, such as TIRF^103^ and HILO microscopy^104^ as well as AFM^105^ and SMLM.^106^ However, all these combined approaches of SIM are applied to study eukaryotic systems^103,104,106^ as opposed to prokaryotic systems which is the main focus of this review.

### 2.3 Multiplexed Super-Resolution Microscopy Approaches

The fundamental principle behind multiplexed super-resolution microscopy is the ability to distinguish and visualize different biomolecules concurrently within the same sample. Multiplexed super-resolution microscopy combines super-resolution imaging approaches, enabling the imaging of multiple targets simultaneously. By labeling different targets with spectrally distinct fluorophores or by sequentially imaging different targets using photoactivatable or photoswitchable strategies, researchers can visualize multiple biomolecules within the same sample with high spatial resolution. The majority of multiplexed super-resolution microscopy techniques, which combine both SIM and SMLM or STED, have been utilized for studying eukaryotic systems.^102^ In the context of prokaryotic systems, multicolor PALM in combination with membrane PAINT and TIRF microscopy approaches have been used to distinctly identify the target molecules of interest within cells.^100,107^ Studies using a combination of SIM and AFM have provided insights into the organization and dynamics of target cellular structures in bacterial cell.^105^ As these technologies continue to evolve, multiplexed super-resolution microscopy is expected to play an increasingly significant role in unraveling the complexities of biological systems at the nanoscale.

## 3 Applications of Single-Molecule Techniques in Genome Maintenance Processes

In this section, we will highlight the outcomes of single-molecule studies using all the in vitro and in vivo imaging techniques discussed in the previous section (Section 2). We will explain the findings in the context of DNA replication, transcription, DNA repair, and recombination, as these cellular processes are crucial for maintaining genome stability within prokaryotic organisms.

### 3.1 DNA Replication

DNA replication is ensures the accurate duplication of genomic information, carried out by the multiprotein complex referred to as the replisome. DNA replication is essential for the transmission of genetic information from one generation to the next and for the functioning of the cell. Single-molecule studies have provided valuable insights into the dynamics and mechanisms of DNA replication by allowing direct observation of individual replisomes reconstituted on a DNA template. This achievement has been accomplished through the use of single-molecule assays based on rolling-circle DNA amplification, which have been well reviewed in Kaur et al.^108^

**Figure 4:**
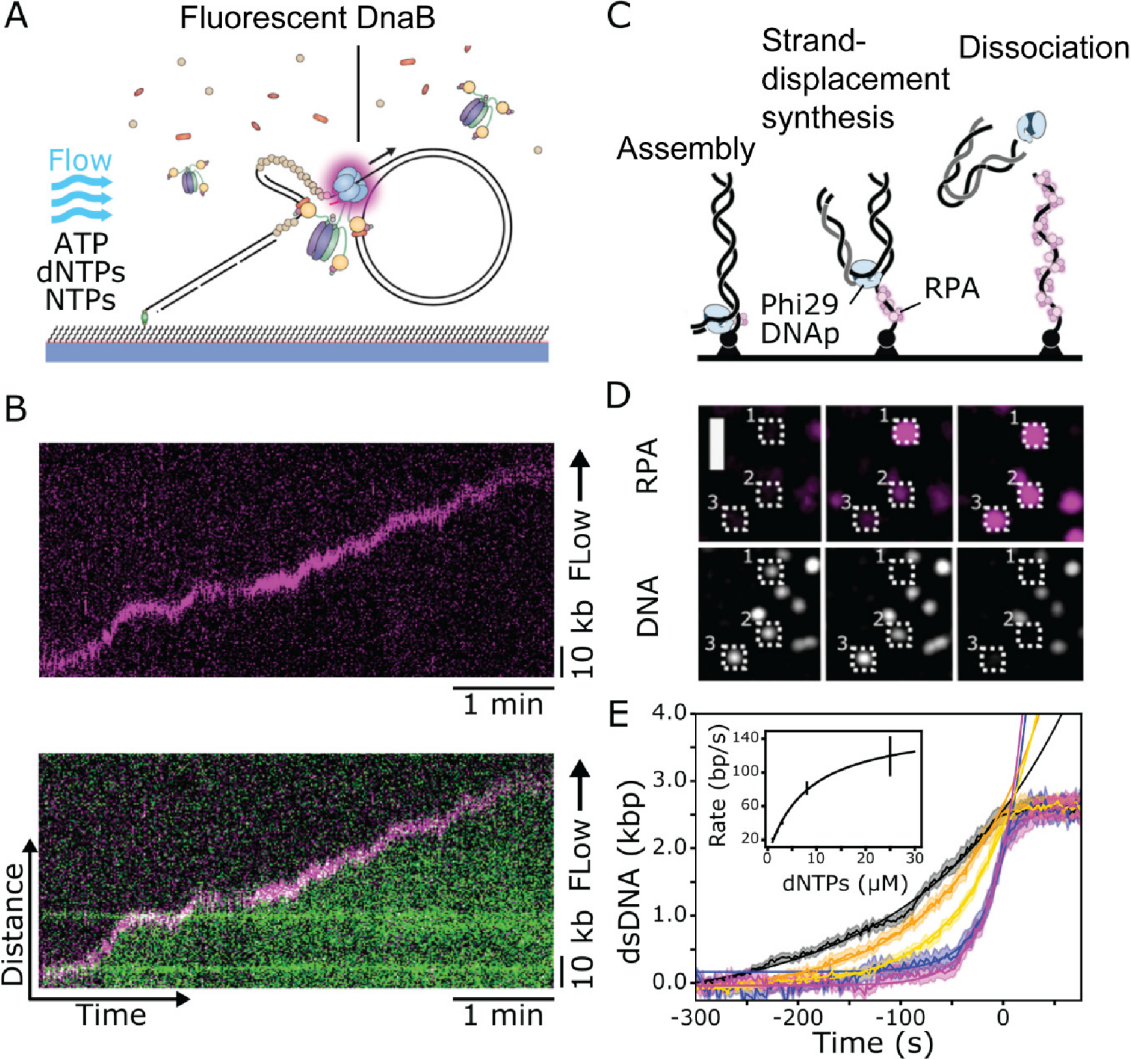
In vitro single-molecule DNA replication assays using TIRF microscopy. (A) Rolling-circle DNA replication by the *E. coli* replisome with fluorescently labeled DnaB helicase. The replisome uses a circular DNA template to produce a long linear stretch of DNA. The synthesized DNA is hydrodynamically stretched using microfluidics. Reproduced with permission from ref (109). Copyright 2021 Oxford University Press. (B) A kymograph produced by single-molecule rolling-circle DNA replication. The top panel (magenta) shows the movement of the DnaB helicase during DNA replication. The bottom panel shows DnaB (magenta) stably associated with the replication fork, ahead of the newly synthesized double-stranded DNA (green). Reproduced with permission from ref (109). Copyright 2021 Oxford University Press. (C) Schematic of simplified single-molecule replication assay for Phi29 DNA polymerase, acting on immobilized linear DNA substrates. After assembly the polymerase synthesizes DNA while simultaneously exposing single-stranded DNA on the opposite strand. The reaction is monitored using the double-stranded DNA dye Sytox orange and a fluorescently labeled single-stranded binding protein (RPA). Reproduced with permission from ref (110). Copyright 2022 Oxford University Press. (D) Montage of TIRF microscopy movie of strand-displacement synthesis. The magenta spots (top row) increase in intensity as ssDNA is produced, while the white spots (bottom) stay constant until the dissociation of the daughter strand. (E) Replication kinetics at different nucleotide concentrations (black: 1 μM, orange: 2 μM, yellow: 3 μM, magenta: 8 μM and blue: 25 μM). The inset shows the extracted rates of DNA synthesis rates.

In 2009, Tanner et al. established an in vitro TIRF microscopy method to visualize individual DNA molecules undergoing rolling-circle DNA replication in real-time.^111^ This technique has enabled precise measurement of rates and processivities (amount of DNA synthesized) of T7 and *E. coli* replisomes. Later, this technique was further developed to include fluorescently labeled proteins, such as DNA polymerases or helicases (Figure 4A). The traditional view was that all components of the replisome remain stably associated within the complex, much like the mechanical parts of an engine.^26,112,113^ However, single-molecule studies revealed that DNA Polymerases, for example, rapidly exchange,^26^ and that components of the replisome can act independently and stochastically.^114^ This plasticity is an inherent property of protein complexes and shows that the “mechanical engine” analogy is of limited use. The dynamic exchange of DNA polymerases has also been observed in living bacterial cells,^26,115,116^ for bacteriophage T7,^117,118^ as well as in eukaryotic systems.^119,120^ It has been proposed that this plasticity allow the replisome to adapt to different cellular stresses and to overcome DNA damage during replication.^121^ Similar to the DNA polymerase exchange phenomenon, single-molecule methodologies using both in vitro and in vivo approaches, have clearly demonstrated the recycling of SSB proteins across multiple Okazaki fragments.^30,122,123^ Additionally, in vivo single-molecule studies have provided substantial insights into the behavior of *E. coli* replisomes within live cells. These studies have demonstrated the ability to track replisome positions by monitoring replication from initiation to termination,^124^ offering real-time insights into the stoichiometry and architecture of the replisome,^125^ within live *E. coli* cells.

However, not all the components of the replisome are recycled or exchanged during DNA replication. Recent in vitro single-molecule studies have shown that the replicative helicase DnaB, an enzyme that catalyzes the unwinding of the parental DNA double helix, remains stably associated at the replication fork for more than 30 min. Figure 4B shows a kymograph, the frames of a movie showing replication arranged in a single image, of DnaB. The magenta line corresponds to a single DnaB helicase traveling with the replication fork as new double-stranded DNA (stained by Sytox orange, green) is synthesized. This finding suggests that the stability of DnaB at the replication fork is crucial for the successful progression of DNA replication. Furthermore, these data suggest that the DnaB helicase forms the interaction hub for other replisome components. Force-based single-molecule studies on the T7 system have shown that the unwinding ability of helicases is sequence dependent and that the rate of unwinding increases when destabilizing forces are present on the replication fork junction,^126^ consistent with the results of ensemble studies on helicase unwinding.^127^ Collectively, these single-molecule observations support the notion of highly dynamic characteristics of replisomal components highlighting the complex interplay between various molecular players involved in the fundamental biological process of DNA replication.

Single-molecule techniques have been used for over two decades to study DNA replication and related processes. However, the number of laboratories contributing such single-molecule studies is very small. The reason for this is the high level of expertise and complicated measurement apparatus required. To enhance the reproducibility of quantitative fluorescence microscopy a simplified method for the study of various enzymes involved in DNA replication transcription and repair was proposed.^110^ Similar to previously described methods it relies on immobilized linear DNA substrates. In contrast to other methods, however, it does not require external forces applied through hydrodynamic flow or magnetic or optical trapping of microparticles. Enzyme activity is quantified by measuring the fluorescence intensity from specific double- and single-stranded DNA markers within diffraction limited spots (Figure 4C). The produced image data is of comparatively low complexity and be analyzed rapidly and in a highly automated fashion. Figure 4D and 4E show replication of individual DNA substrates by the phage Phi29 DNA polymerase. Despite the low complexity the assay is still capably of capturing single-molecule dynamics, yet hundreds of molecules are studied simultaneously. This improves the statistical power and therefore the reproducibility. The method has also been shown to be adaptable to a wide range of DNA modifying enzymes involved in genome maintenance, such as helicase and exonucleases.

### 3.2 Transcription

**Figure 5:**
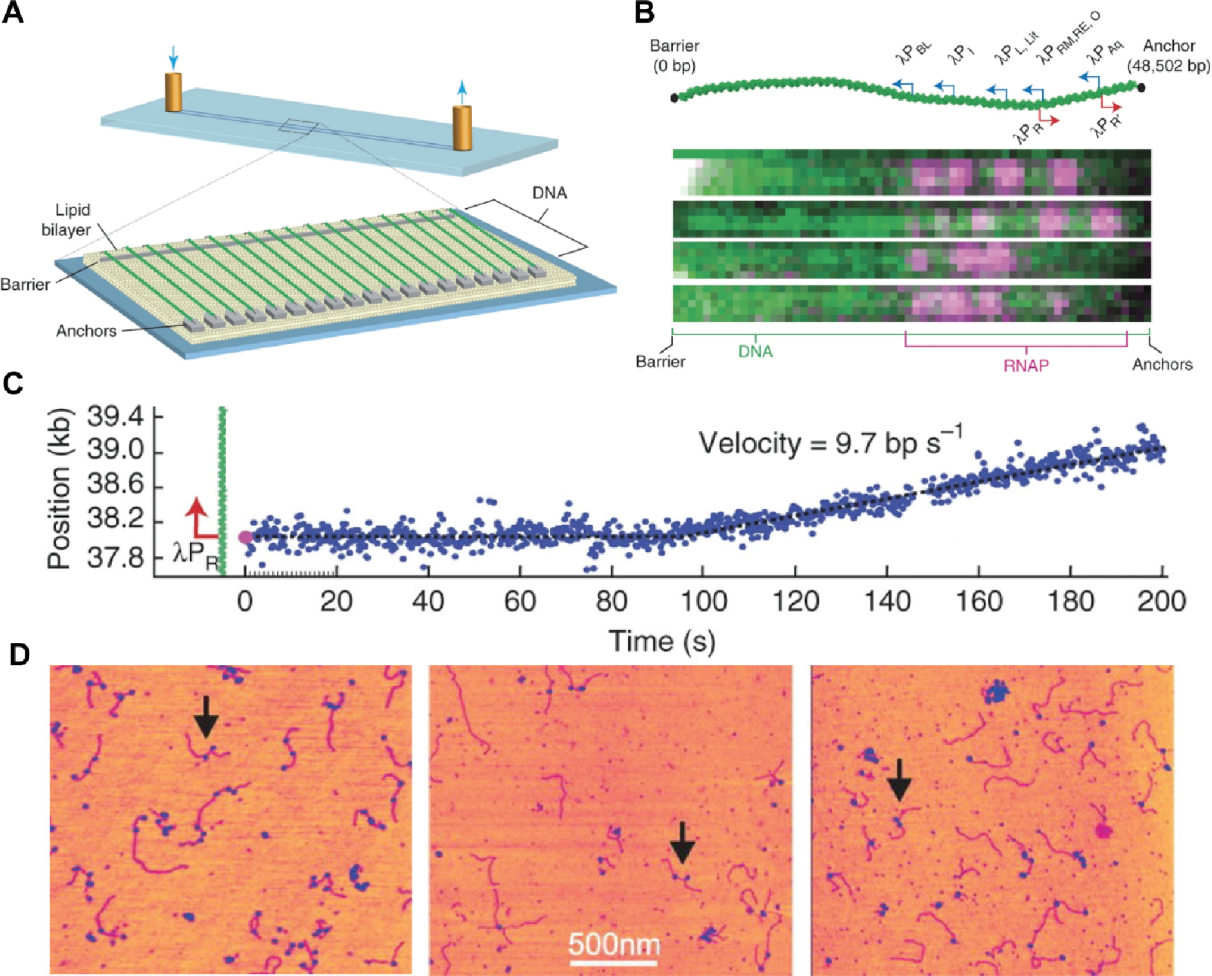
In vitro single-molecule transcription assay using TIRF and HS-AFM microscopy. (A) Schematic representation of DNA curtain assay used for promoter-specific binding by RNAP. Reproduced with permission from ref (131). Copyright 2012 Springer Nature. (B) RNAP-bound (magenta) to specific promoter sites on 48.5-kb long λ-phage genome. Reproduced with permission from ref (131). Copyright 2012 Springer Nature. (C) Representative transcription rate after initiating transcription from the λPR promoter (9.7 bp/s). Reproduced with permission from ref (131). Copyright 2012 Springer Nature. (D) Atomic force microscopy images of open promoter complexes (OPCs) formed in the absence of NTPs (left image), stalled elongation complexes (SECs) formed in the presence of ATP, GTP, and UTP (middle image) and elongation complexes (ECs) formed upon the addition of NTPs (right image). The RNAP are indicated as globular features (blue) on the DNA template (magenta) and the arrows denote DNA molecules harboring two RNAP. Reproduced with permission from ref (60). Copyright 2006 Oxford University Press.

Transcription involves the synthesis of RNA from a DNA template by RNA polymerase (RNAP). The process comprises three stages: initiation,^128^ elongation,^129^ and termination.^130^ In 2013, Wang et al. used a DNA curtain assay, whereby individual DNA molecules are stretched and immobilized in parallel on a surface, forming a “curtain” of DNA strands (Figure 5A). They show that the promoter search mechanism by *E. coli* RNAP is dominated by 3D-diffusion rather than 1D-facilitated diffusion.^131^ This result demonstrated that there is a higher probability of RNAP directly binding to dispersed promoter sites through 3D collisions. Additionally, these stretched DNA templates provide an ideal platform for the observation of colocalization and movement of RNAP along the DNA template in real-time (Figure 5B). This approach also allows measurement of dynamic transcription rates, complementing the insights gained from studies on rolling-circle DNA replication^26,111,132^ (Figure 5C).

Furthermore, AFM techniques have been employed to study collision events between RNAP in convergent transcription.^60^ Convergent transcription in bacteria refers to a condition where two adjacent genes are transcribed in opposite directions, causing the RNAPs to move toward each other. This results in overlapping or converging transcriptional units, leading to the formation of antisense RNAs and causing premature transcription termination, affecting gene regulation and genome stability.^133^ By using AFM, real-time visualization of collisions between transcription machineries has shown that one RNAP typically forces the other RNAP to backtrack along the template after collision.^60^ RNAP backtracking refers to the process where RNAP moves backward along the DNA template, resulting in the displacement of the nascent RNA transcript from the active site.^134,135^ RNAP backtracking plays a crucial role in transcriptional regulation by controlling gene expression in response to regulatory signals or bypassing transcriptional roadblocks such as DNA lesions, protein-bound DNA regions, or stable RNA secondary structures like R-loops.^135^ Moreover, the AFM study also demonstrated that RNAP bound to DNA can exist in three different form, open promoter complexes (OPC) or stalled elongation complexes (SEC) or elongation complexes (EC), depending on the presence or absence of NTPs (Figure 5D).^60^

Likewise, HS-AFM has been employed to directly visualize the molecular movement of a bacteriophage T7 RNAP, including its DNA binding, sliding, and RNA synthesis during the transcription process.^136^ Altogether, these studies have highlighted the complexity involved in tracking the movement of individual RNAP molecules along the DNA during transcription initiation and elongation. These findings challenge the traditional view of transcription as a smooth and continuous process.^137^ Instead, they show that transcription is a highly dynamic process, which undergoes 3D-diffusion during the initial stages while searching for promoter sites, followed by 1D-sliding during the transcription elongation phase along the DNA template. These revelations underscore the multifaceted nature of transcriptional regulation and provide a solid foundation for further exploration into the molecular mechanisms governing gene expression.

### 3.3 DNA Repair

The genome of any organism is continuously subject to damaging processes. This, not only includes the impact of various chemical agents and radiation,^138^ but even cellular processes like DNA replication or transcription can occasionally lead to DNA damage by altering the secondary DNA structures.^139,140^ DNA repair processes include a range of enzymatic pathways to repair otherwise toxic alterations of DNA. Single-molecule studies on bacterial DNA repair have unraveled how individual molecules participate in the repair of damaged bacterial DNA. The ability to observe and dissect individual repair events in real-time has provided unprecedented insights into the dynamic nature of DNA repair processes. One of the prominent areas of single-molecule research in bacterial DNA repair involves the study of nucleotide excision repair (NER), a versatile pathway that addresses a broad spectrum of DNA lesions induced by UV radiation and chemical agents. This process is facilitated by UvrA, UvrB, UvrC, and UvrD repair factors.^141^ Briefly, UvrA acts as a sensor for DNA damage through increased binding affinity for damaged DNA.^142^ Subsequently UvrB binds to DNA-bound UvrA, in turn recruiting UvrC which creates incisions on either side of the lesion. Next, UvrD, a processive DNA helicase capable of removing DNA-bound proteins^110^ is thought to remove the damaged strand. Finally, the resulting single-stranded gap can be filled in by a DNA polymerase and ligated by a DNA ligase.

**Figure 6:**
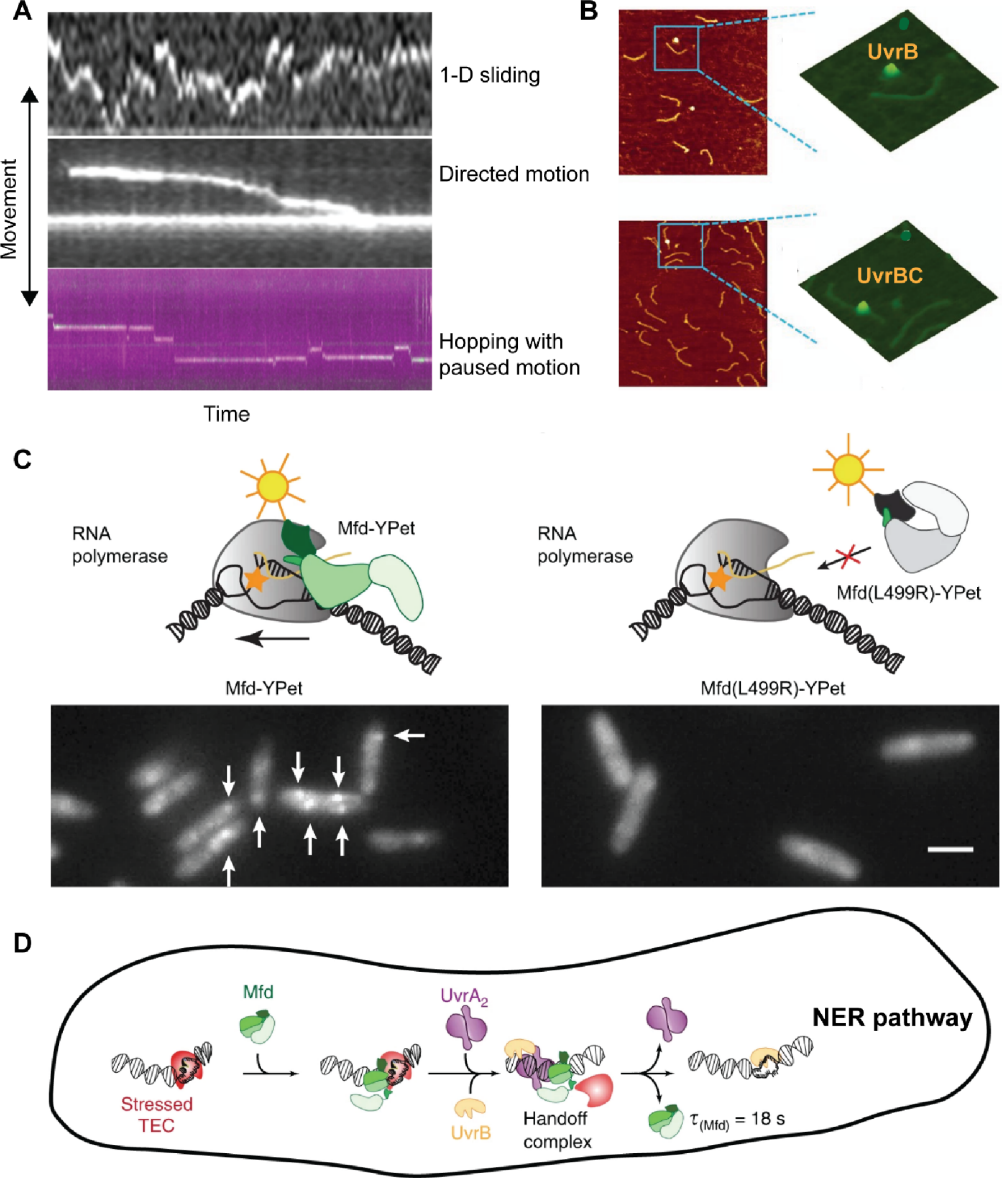
Single-molecule visualization of DNA repair factors involved in NER and TCR pathways using AFM and HILO. (A) AFM movies showing different movement patterns of the UvrAB complex on the undamaged DNA template, including one-dimensional diffusion (top), directed motion (middle), and DNA hopping with a regular interval of paused motion (bottom). Reproduced with permission from ref (143). Copyright 2010 Elsevier. (B) AFM images showing that UvrB molecules, in the absence of UvrC, cannot bind to DNA (top), whereas in the presence of UvrC, UvrB binds to DNA, indicating that the interaction between UvrB and UvrC activates the DNA-binding activity of UvrB. Reproduced with permission from ref (61). Copyright 2013 Oxford University Press. (C) Schematic of wild-type Mfd-Ypet and Mfd (L499R)-Ypet mutant recruited to RNA polymerase stalled at bulky lesions (orange star) on the transcribing DNA strand (top). Fluorescence images showing *E. coli* cells expressing well-defined Mfd-YPet foci (indicated by the white arrow), whereas cells expressing the Mfd (L499R)-Ypet mutant were unable to form foci (bottom), suggesting that Mfd is associated with RNAP in the absence of DNA damage. Scale bar, 2 μm. Reproduced with permission from ref (144). Copyright 2018 Springer Nature. (D) Proposed in vivo model of Mfd-UvrA2-UvrB handoff complex formation suggests a dynamic interplay between Mfd, UvrA2, and UvrB proteins during the recognition and repair of DNA lesions. (For interpretation of the references, the reader is referred to the web version of this article). Reproduced with permission from ref (145). Copyright 2020 Springer Nature.

Single-molecule techniques using AFM have shown the interactions between UvrA and UvrB complexes, revealing how they recognize DNA lesions and facilitate the recruitment of UvrC to initiate the repair process.^61,143,146^ Initially, the stable UvrAB complexes exhibit a range of motions on the DNA, including one-dimensional diffusion, ATP-dependent directed motion, paused motion, and DNA hopping through rapid excursions to new positions on the DNA (Figure 6A).^143^ Subsequently, UvrC interacts with UvrB to form stable UvrBC complexes (Figure 6B).^61^ A special type of NER occurs when DNA damage is encountered by transcribing RNAP rather than UvrA. This process is referred to as transcription-coupled repair (TCR).^147,148^ Through a single-molecule live-cell imaging approach, Ho et al. in 2018 found that DNA repair protein Mfd stably associates with stalled transcription elongation complexes, whereas Mfd (L499R) mutant (lacks the ability to interact with β-subunit of RNAP) are unable to interact with RNAP (Figure 6C).^144^ The authors also demonstrated that the residence time of Mfd decreases in the presence of UvrA, indicating potential involvement of UvrA in resolving Mfd-RNAP complexes on DNA.^144^ Later, it was revealed that the binding lifetime of UvrA with Mfd during TCR is governed by UvrB, suggesting a central role of UvrB in resolving Mfd-UvrA intermediates at the RNAP stall site.^149^ Furthermore, Ho et al. in 2020 showed that UvrA-mediated recruitment of UvrB on DNA promotes the dissociation of Mfd and UvrA, completing the handoff to downstream nucleotide excision repair factors (Figure 6D).^145^

**Figure 7:**
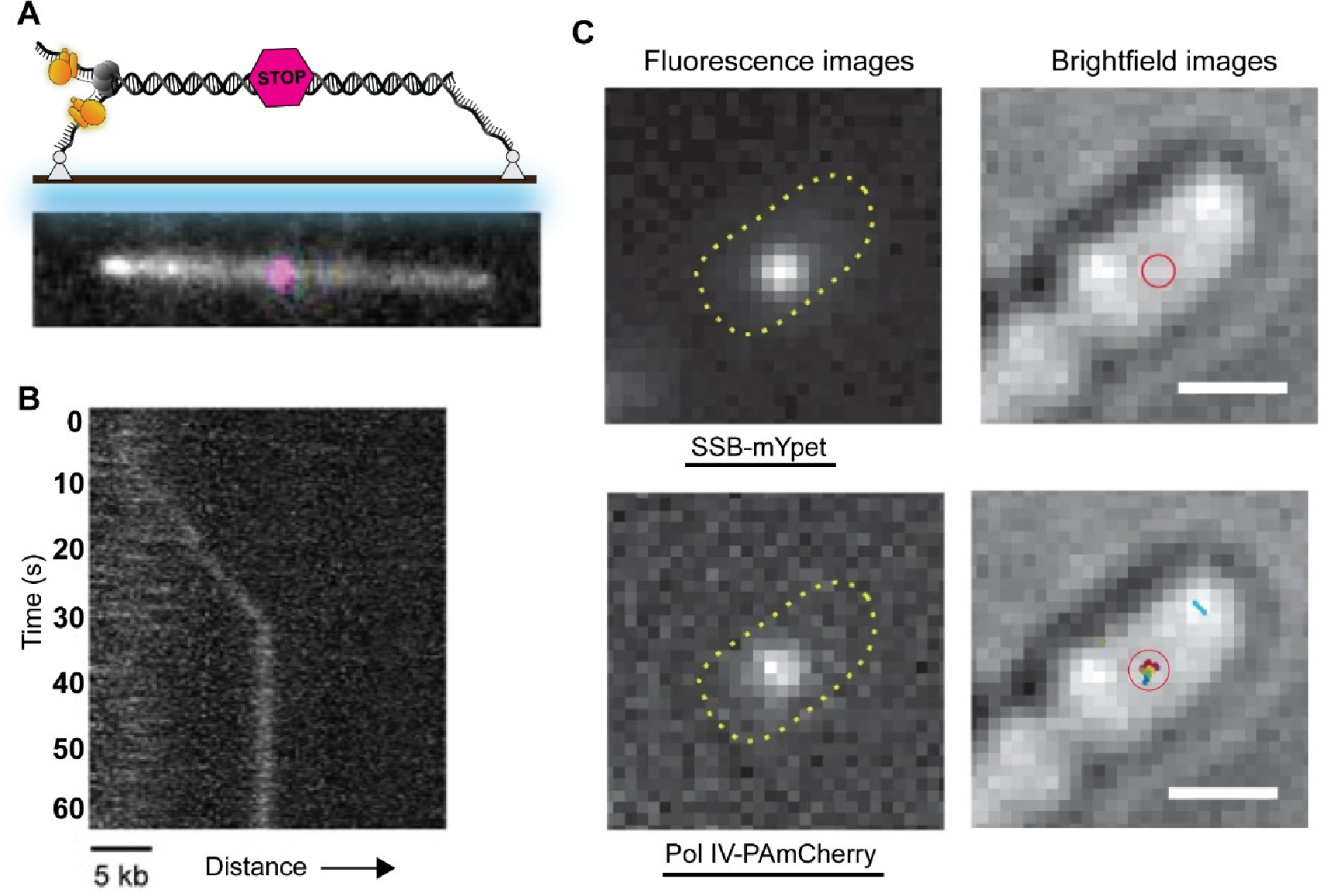
Single-molecule visualization of in vitro stalled replication and formation of in vivo SSB-bound ssDNA intermediates after encountering DNA lesions. (A) Schematic representation of a 36-kb linear DNA template with a site-specific lesion (magenta) in the middle (top). Example showing a 36-kb linear DNA template with a Cy5 fluorophore marker at the position of a site-specific roadblock (Bottom). Reproduced with permission from ref (152). Copyright 2022 Elsevier. (B) Example kymograph showing stalling of DNA replication at the site of a CPD lesion. Reproduced with permission from ref (152). Copyright 2022 Elsevier. (C) Example fluorescence images (left) of an *E. coli* cell expressing fluorescent SSB-mYpet focus (top panel) and fluorescent Pol IV-PAmCherry focus (bottom panel). The corresponding brightfield images (right) show the position of the SSB focus (red circle, top panel) and all detected Pol IV tracks (colored dots within the red circle, bottom panel), suggesting that SSB enriches Pol IV at lesion-stalled replication forks. Scale bar indicates 1 μm. Reproduced with permission from ref (157). Copyright 2022 Springer Nature.

Encountering DNA lesions or protein roadblocks by the replisome can lead to stalling of the replication fork and subsequently to incomplete replication and DNA breaks.^150,151^ The mechanisms by which the replisome can overcome DNA lesions and how genomic integrity is maintained in those situations remain incompletely understood. In 2022, Kaur et al. developed a 36-kb linear DNA template to visualize encounters between the replisome and a single DNA lesion (Figure 7A).^152^ The study shows stalling of individual replisomes upon encountering a UV induced lesion. This finding is in stark contrast to previous ensemble studies that showed efficient bypass of lesions by the *E.coli* replisome (Figure 7B).^153^ Likewise, in 2005, Indiani et al. proposed a “toolbelt” model that suggested the simultaneous binding of both DNA Polymerase III and the translesion DNA Polymerase IV to the DNA sliding β-clamp, a ring-shaped dimeric protein that encircles the DNA and provides processivity to DNA replication.^154^ The authors indicated that polymerase switching is regulated when the high-fidelity DNA Polymerase III stalls after encountering a DNA lesion, allowing DNA Polymerase IV to proceed with the translesion bypass process, ensuring efficient replication. However, single-molecule in vivo studies challenged this model and showed that DNA Polymerase III and the translesion DNA Polymerase IV do not form a stable complex with the β-clamp, and therefore, the clamp does not function as a molecular toolbelt.^155,156^ These observations indicate that a different molecular mechanism is at play for the recruitment of TLS polymerases.

Recent in vivo single-molecule studies by Chang et al. 2022 proposed that SSB might fulfill this role. They propose that SSB associated with stalled replication forks at the site of DNA lesions, facilitates access of Polymerases IV.^157^ The study demonstrated that, fluorescent Polymerase IV was colocalized with fluorescent SSBs at stalled replisomes (Figure 7C).^157^ The authors proposed that SSB-bound ssDNA intermediates create a platform where repair and recombination factors are recruited to initiate and facilitate the repair of damaged DNA.^158−160^

However, the mechanism by which SSB moves within the crowded cellular environment remains unclear. In vitro single-molecule studies have suggested that within crowded cellular environment, SSB diffuses via sliding mechanism on the DNA to interact with SSB interacting proteins.^161^ The interaction of SSB-bound ssDNA with various DNA repair and recombination factors is a fundamental step in ensuring the efficient and accurate restoration of genomic integrity in response to DNA lesions or damage.

In general, single-molecule investigations into bacterial DNA repair have played a vital role in elucidating the complex mechanisms of identifying and repairing DNA damage. These studies have not only provided detailed insights into the molecular events involved in DNA repair but have also paved the way for a more comprehensive understanding of how bacteria maintain genomic integrity via various subpathways in the face of various DNA lesions. The knowledge obtained from these findings not only enhances our understanding of fundamental biological DNA repair processes but also lays a foundation for the development of targeted therapies for diseases associated with DNA damage or for combating bacterial infections.

### 3.4 DNA Recombination

**Figure 8:**
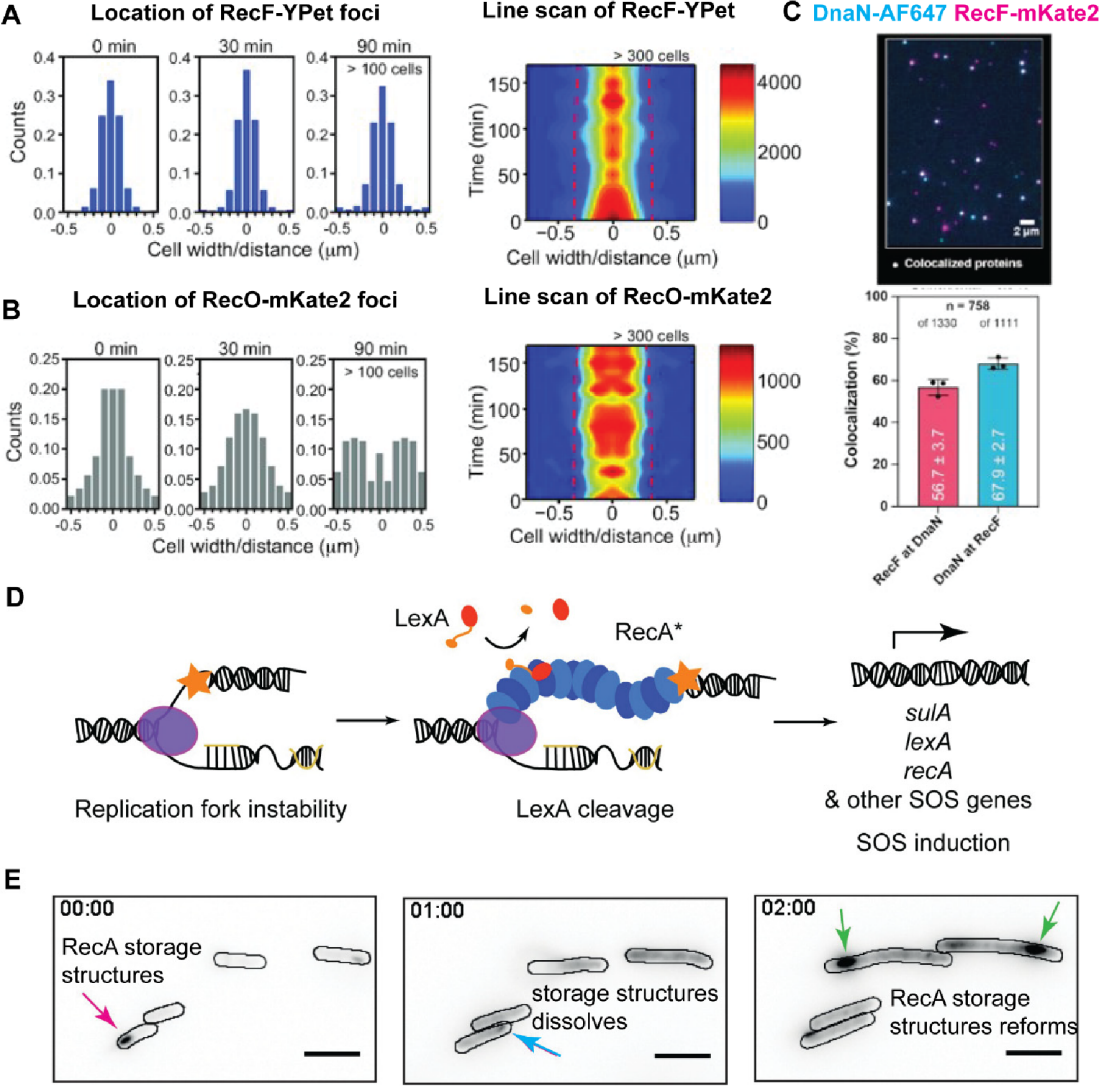
Spatiotemporal characterization of different recombination factors involved in recombinational DNA repair within live *E. coli* at the single-molecule level. Distribution of (A) RecF-YPet and (B) RecO-mKate2 foci before and after DNA damage occupying distinct positions within cells. Reproduced with permission from ref (75). Copyright 2019 Oxford University Press. (C) Single-molecule colocalization of RecF-mKate2 (magenta) with DnaN-AF647 (cyan) suggests RecF interaction with DnaN, a component of the *E. coli* replisome. Reproduced with permission from ref (169). Copyright 2023 Oxford University Press. (D) Schematic showing the formation of ssDNA-bound RecA* filaments at sites of stalled replication forks. The activated RecA* cleaves LexA repressor proteins to induce the SOS response within the cell. Reproduced with permission from ref (77). (E) Time-lapse images of *recA-gfp* cells exhibiting RecA-GFP foci of various morphologies at different stages during the SOS response upon exposure to UV. Magenta arrow indicate foci that are present before damage, and blue arrow indicate foci that disappear during the SOS response. Green arrows indicate foci that reappear after recombinational DNA repair. Reproduced with permission from ref (77).

Recombinational DNA repair is a crucial cellular process that addresses double-strand breaks (DSBs), one of the most severe forms of DNA damage. One central aspect of recombinational DNA repair is homologous recombination, carried out by the RecA recombinase protein.^162−165^ This process involves the exchange of genetic material between homologous DNA strands by invading the undamaged DNA strands with homologous sequences. Single-molecule studies have shown that, prior to homologous recombination, various recombination mediator proteins (RMPs) facilitate the loading of RecA onto the DNA, promoting homologous recombination.^33^ For a detailed review of how RMPs promote homologous recombination in various subpathways see Henry et al. 2021 and Whinn et al. 2021.^67,92^ One important pathway in facilitating recombination the RecFOR pathway. It is mediated by the RecF, RecO, and RecR proteins, with their proposed function being to facilitate the loading of RecA onto single-stranded DNA.^166^ In vitro studies have shown that the RecOR complex promotes RecA nucleation on the SSB-coated ssDNA by removing the SSB, whereas the RecFR complex binds to dsDNA and can act as a barrier to RecA filament extension.^167,168^ Single-molecule live-cell imaging using chromosomally fused fluorescent RecF and RecO has further revealed that RecF and RecO occupy distinct spatiotemporal locations within bacterial cells following DNA damage.^75^ RecF is found to colocalize near the replisome, whereas the RecO-occupied region is close to the cell membrane (Figure 8A and 8B).^75^ In a more recent study by Henry et al. in 2023, it was proposed that RecF’s interaction with the β-clamp potentially triggers the creation of postreplication DNA gaps, which are then processed by the RecFOR system via RecA-mediated recombination (Figure 8C).^169^

Besides recombination, RecA also orchestrates the cellular response to DNA damage via the bacterial SOS response.^170^ The SOS response is a bacterial damage response system triggered by the accumulation of ssDNA when replication stalls at the site of a lesion, while the replicative helicase continues unwinding DNA.^170^ RecA senses the presence of ssDNA regions and binds to them, forming RecA nucleofilaments. In the presence of ATP, RecA nucleofilaments are activated (sometimes referred as RecA*), facilitating the autocatalytic cleavage of the LexA repressor protein (a protein that inhibits the transcription of SOS genes involved in DNA repair and mutagenesis).^170^ The derepression of LexA in turn leads to the expression of a set of SOS genes involved in various DNA repair mechanisms, such as nucleotide excision repair, recombinational repair, and error-prone DNA polymerases (Figure 8D).^170^ Single-molecule live-cell imaging has revealed that under normal metabolic conditions, RecA is predominantly confined to “storage structures” within the cell.^77^ When DNA damage occurs, these storage structures disassemble, and RecA forms elongated bundles on DNA (Figure 8E).^77^ RecA storage structures reassemble upon the completion of repair.^77^

While single-molecule live cell imaging using HILO offers advantages in terms of real-time visualization and spatial organization of repair processes compared to ensemble studies, it cannot overcome the diffraction limit, which imposes a resolution barrier on the detection of molecular events. Consequently, it may not provide the level of detail necessary to resolve individual biomolecular interactions or structural features at the nanoscale. To address this limitation and achieve higher resolution imaging, alternative techniques such as super-resolution microscopy have been developed. These methods surpass the diffraction limit by employing various strategies to localize individual fluorophores with nanometer-scale precision, enabling the visualization of molecular structures and dynamics with unprecedented detail. Thus, while single-molecule live cell imaging using HILO has significantly advanced our understanding of repair processes, overcoming the diffraction limit remains a critical challenge in the field of cellular imaging.

### 3.5 Outcomes from the Super-Resolution Microscopy Approach

**Figure 9:**
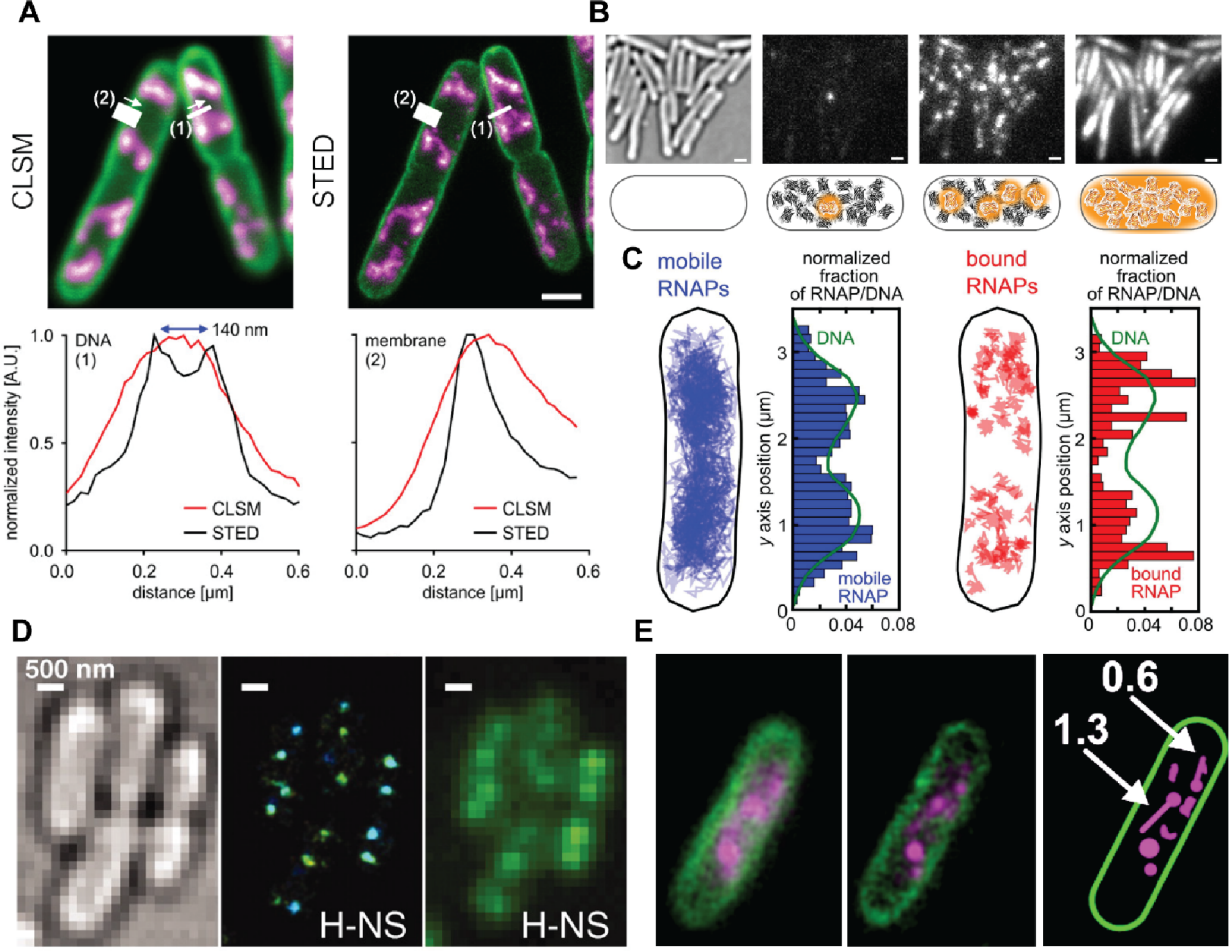
Examples of fluorescent live-cell images of *E. coli* acquired using super-resolution microscopic techniques. (A) Two-color CLSM (left) and STED (right) images of *E. coli* DNA (magenta) and membrane (green) as shown in the panel. The cross-sections of DNA filaments (1) and membrane (2) show enhancement of resolution by STED (bottom panel). Reproduced with permission from ref (171). Copyright 2018 American Chemical Society. (B) Transmitted light microscopy image, single photoactivated fluorophore (DNA polymerase I tagged with Pam-cherry) within one cell, image of multiple photoactivated fluorescent molecules within the cell, and integrated fluorescence images from a PALM movie (from left to right). Schematics are shown underneath each panel. Reproduced with permission from ref (93). Copyright 2014 Creative Commons Attribution (C) Example distribution of mobile RNAP (blue lines/bars) and bound RNAP (red lines/bars) trajectories matched with the distribution of DNA (green line). Reproduced with permission from ref (94). Copyright 2015 National Academy of Sciences. (D) Brightfield image (left), 3D STORM image (middle), and conventional fluorescence image (right) showing compact H-NS clusters in the nucleoid within live cells. Reproduced with permission from ref (97). Copyright 2011 American Association for the Advancement of Science. (E) Wide-field epifluorescence and SIM images showing distinct SSB structures within live cells exposed to DNA damage. Reproduced with permission from ref (172). Copyright 2019 Molecular Biology Society of Japan and John Wiley & Sons Australia.

By employing super-resolution microscopy, researchers have been able to explore the intricacies of prokaryotic genome maintenance at a level of detail not achievable with traditional single-molecule microscopy techniques. These techniques have revolutionized the study of protein–DNA interactions within living cells. A recent STED microscopy approach has shown that the use of fluorophores that reversibly bind to their target structures allows photobleached fluorophores to be replaced through exchange with intact fluorophores from the solution, circumventing limitations from photobleaching.^171^ The multiple structures of the whole bacterial cell membrane and DNA were imaged using Nile Red and JF646-Hoechst as exchangeable labels with a long acquisition time and high laser powers without signal loss (Figure 9A).^171^ The cross-sections of DNA filaments (1) and membrane (2) showed enhancement of resolution by STED compared to images obtained from confocal laser scanning microscopy (CLSM) (bottom panel of Figure 9A). Using this approach the authors were able to construct the multicolor 3D-STED images of live bacterial cell and eukaryotic HeLa cells.^171^ This is important because it demonstrates the potential of STED microscopy to visualize cellular structures at nanoscale resolution within live cells in real-time, thereby enhancing our understanding of cellular organization and function.

High-resolution microscopy setups, using PALM, have allowed visualization, quantification, and tracking of protein–DNA interactions in living *E. coli* bacteria. In 2014, Uphoff et al. showed that the photoactivatable fluorescent protein PAmCherry genetically fused to DNA polymerase I within cells can be stochastically activated using a short pulse of soft UV-light and tracked over multiple frames until photobleaching occurs.^93^ The PALM images of cells are constructed by integrating the subsets of labeled DNA polymerase I molecules activated at a given time to determine their positions in a sequential manner, independently of the total concentration of labeled molecules within the cell (Figure 9B). By analyzing the diffusion patterns of individual proteins over time, transient binding events between a protein and DNA can be identified based on a reduction in the apparent diffusion coefficient of the protein. This binding data provides a quantitative measure of the DNA-binding activity and substrates of the protein of interest in live cells.^93^ Additionally, by combining single-particle tracking analysis with PALM allows observing the spatial organization of the molecules of interest within the cell. For instance, Stracy et al. 2015, indicated that mobile RNAPs extensively explore the nucleoid during their promoter search, spending the majority of their time in nonspecific interactions with DNA, whereas specifically bound RNAPs are found to form dense clusters predominantly at the nucleoid periphery, suggesting that transcription can cause spatial reorganization of the nucleoid, leading to the movement of highly transcribed genes out of the bulk of DNA as transcription levels increase.^94^ The distribution of mobile RNAPs closely resembles the distribution of DNA, while the bound RNAPs within cells are more heterogeneous (Figure 9C).^94^

Likewise, STORM has been used to investigate the global organization of the bacterial chromosome in live *E. coli* cells. In 2011, Wang et al. showed that four nucleoid-associated proteins (NAPs)—HU, Fis, IHF, and StpA—are largely scattered throughout the nucleoid.^97^ Meanwhile, the silencer protein H-NS forms two compact clusters per chromosome driven by the oligomerization of DNA-bound H-NS.^97^ A 3D STORM image of live *E. coli* cells expressing the photoactivatable fluorescent protein mEos2 fused to H-NS revealed compact H-NS clusters in the nucleoid region with enhanced resolution compared to conventional fluorescence cell images (Figure 9D).^97^

With the help of the super-resolution technique SIM, Zhao et al. 2019, showed the subcellular localization of SSB proteins within *E. coli* cells under normal and DNA-damaged conditions.^172^ The results showed that under normal conditions, SSB tagged with GFP localizes to the inner membrane of the cell, but after DNA damage (induced by mitomycin C), SSB rapidly disengaged from the membrane within 5 min and became associated with the nucleoid. SIM images revealed distinct elongated SSB structures within live cells exposed to DNA damage compared to wide-field epifluorescence images (Figure 9E).

### 3.6 Outcomes from Multiplexed Super-Resolution Microscopy Approach

**Figure 10:**
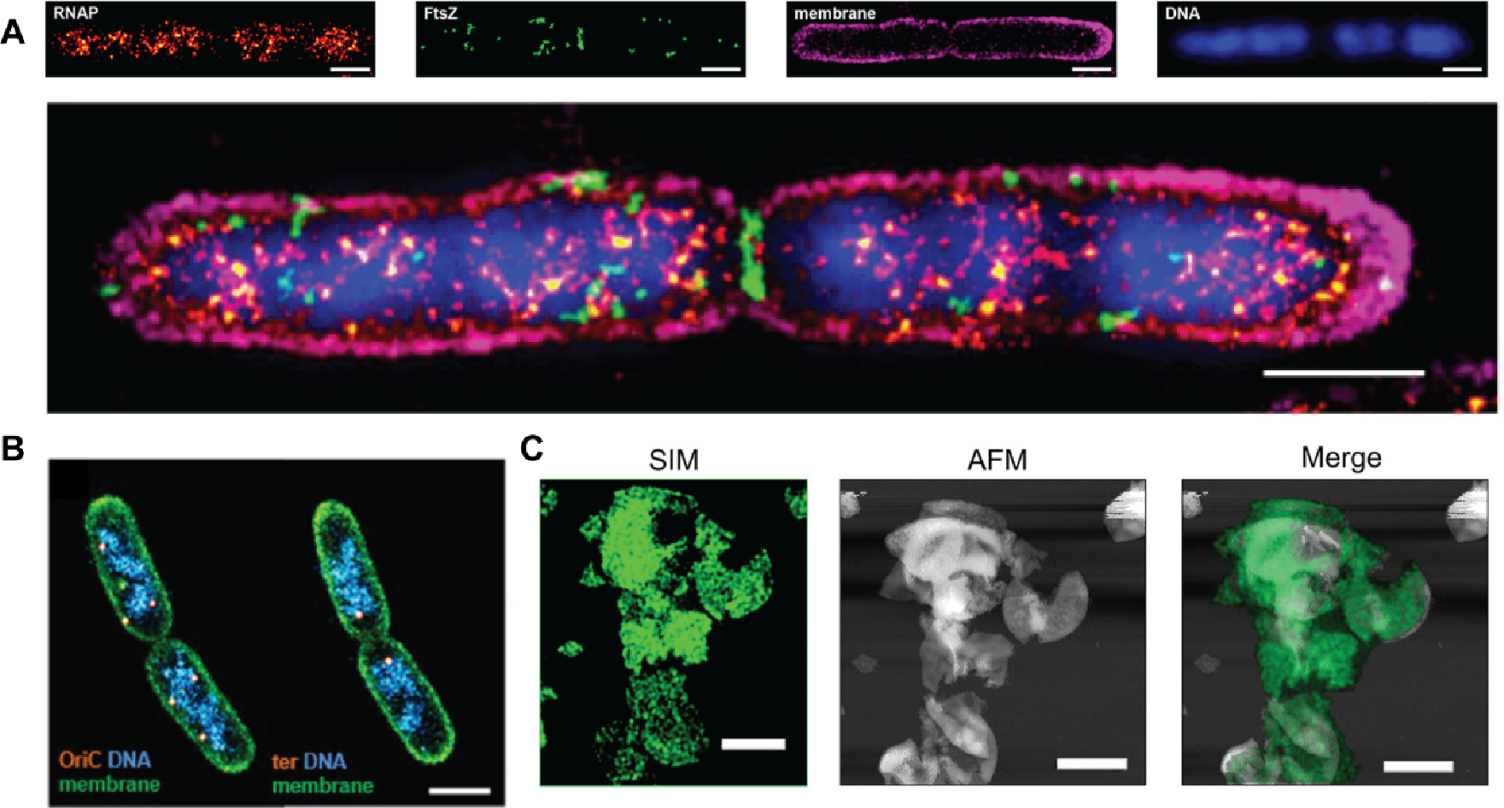
Multicolor fluorescent images were acquired using multiplexed super-resolution techniques. (A) Multicolor imaging workflow sowing PALM image of RNAP and FtsZ, membrane PAINT image, and SYTOX-stained DNA image with TIRFM in *E. coli* cells (top). Colocalization of the transcription machinery and the nucleoid, coimaged with FtsZ and the membrane in *E. coli* (bottom panel). Scale bare, 1 μm. Reproduced with permission from ref (107). (B) Multicolor PALM image of genetic loci (yellow), the nucleoid DNA (blue), and the membrane (green) in fixed *E. coli*. Scale bare, 1 μm. Reproduced with permission from ref (100). Copyright 2018 Springer Nature. (C) SIM and AFM merged images of Mre proteins involved in the cell wall synthesis of *B. subtilis*. Scale bar, 2 μm. Reproduced with permission from ref (105). Copyright 2021 American Chemical Society.

Combining different super-resolution microscopy techniques or with conventional single-molecule imaging approaches has further enabled researchers to visualize multiple molecular components involved in genome maintenance simultaneously. For example, labeling different proteins or DNA structures with distinct fluorophores enables the observation of their spatial organization within the prokaryotic cell. In 2017, Virant et al. introduced a novel multicolor imaging strategy using PALM combined with other correlative imaging techniques like membrane PAINT and TIRF to show the spatial organization of RNAP, FtsZ protein, cell membrane, and DNA within live *E. coli* cells (Figure 10A).^107^ Multicolor imaging combining PALM with PAINT has shown the spatial localization of the origin of replication using a ParB tagging system within the nucleoid engulfed within the cell membrane of *E. coli* (Figure 10B).^100^ Additionally, Tank et al. 2021, combined STORM and atomic force microscopy (AFM) to map peptidoglycan synthesis in *Bacillus subtilis* during growth and cell division.^105^ During septation (or division), STORM revealed cell-wall synthesis occurs across the developing septum, suggesting a two-stage process of initial leading-edge deposition followed by pore filling. AFM shows septa contain large pores that get infilled over time. During elongation, STORM/SIM visualize periodic striped patterns of synthesis associated with MreB proteins. Correlating super-resolution SIM and AFM provided insight into how bacterial cells synthesize their complex cell wall architectures (Figure 10C).^105^

Altogether, the advent of super-resolution microscopy techniques such as PALM, STORM, SIM, and STED has revolutionized the visualization of molecules of interest within living cells. As technology continues to evolve, super-resolution microscopy is poised to significantly contribute in unraveling the complexities of cellular biology at the nanoscale. While efforts have been made to adapt super-resolution techniques for live-cell imaging, challenges remain in achieving high-resolution imaging of dynamic cellular processes in real-time without compromising cell viability or image quality. Moreover, the field is still new in terms of studying complex molecular mechanisms associated with genome maintenance processes.

## 4 Conclusions and Perspective

In this review, we described common single-molecule techniques used for the study of the molecular mechanisms of prokaryotic genome maintenance. Over the past decades, single-molecule techniques have allowed a transition from ensemble analysis to observation of individual molecules and molecular processes. Currently, we have reached a point where continuously evolving single-molecule techniques can be employed to investigate more complex and previously unexplored mechanisms. These may include replication-transcription conflicts and different recombination subpathways associated with the DNA repair mechanism, all of which are essential for cell function and survival. Given the limitless complexity of the biomolecular processes involved in genome maintenance within the physiological environment of the cell, the use of cutting-edge super-resolution microscopy techniques has further pushed the boundaries of resolution and precision, enabling scientists to map and monitor target molecules individually or collectively at the single-molecule level.

Moreover, single-molecule techniques are increasingly being used to study genome maintenance processes in eukaryotes, expanding our understanding of the intricate molecular mechanisms governing fundamental biological processes like DNA replication^119,173−176^ and transcription.^177,178^ This has allowed for the revelation of new mechanisms underlying genome dynamics, cellular function, and disease pathology, with potential applications, from foundational research to the design of innovative therapeutic strategies in drug development.

However, single-molecule approaches still come with challenges associated with photobleaching and phototoxicity from fluorophores, potentially affecting the behavior of biological molecules. Furthermore, super-resolution techniques require expensive and complex setups and extensive expertise in data analysis, which often limits throughput and therefore the statistical power of many studies. Additionally, while super-resolution techniques offer improved spatial resolution, temporal resolution may be compromised. Limited observation times necessitate high-speed imaging to capture dynamic processes more accurately, as some biological events may occur on timescales that exceed the practical limitations of current single-molecule techniques.

Acknowledging these challenges and actively working to address them through technological advancements, improved methodologies, and standardized protocols^110^ will be essential for maximizing the potential of single-molecule studies in advancing our understanding of genome maintenance processes.

## Vocabulary Terms

TIRF microscopy: Total internal reflection fluorescence microscopy is an advanced single-molecule imaging technique that uses an evanescent wave to selectively illuminate and excite fluorophores in a region near the glass-water interface, enabling high-contrast visualization of fluorophores near the sample surface
HILO microscopy: Highly inclined and laminated optical sheet microscopy is a single-molecule imaging technique that uses a thin, highly inclined sheet of light that illuminates a specimen, reducing background fluorescence and enhancing contrast for high-resolution imaging
AFM: Atomic force microscopy is a nanoscale imaging technique that uses a cantilever with a sharp tip to scan a sample’s surface, producing detailed topographical maps of the specimen
STED: Stimulated emission depletion microscopy is a super-resolution imaging technique that uses a combination of excitation and depletion laser beams to selectively deplete fluorescence around a focal point, achieving higher resolution by confining fluorescence to a subdiffraction-limited volume
SMLM: Single-molecule localization microscopy is a super-resolution imaging technique that achieves high spatial resolution by precisely localizing individual photoactivatable or photoswitchable fluorescent molecules, enabling detailed visualization of structures beyond the diffraction limit
PAINT: Points accumulation for Imaging in Nanoscale Topography (PAINT) microscopy is a super-resolution imaging technique that relies on transient binding events between fluorescently labeled probes, which are continually introduced to the sample, for precise localization of individual molecules
SIM: Structured illumination microscopy is a super-resolution imaging technique that uses patterned illumination to create moiré fringes, allowing high-resolution imaging of samples beyond the diffraction limit
